# Abnormal Dendritic Cell-poiesis in Patients With Lower-risk Myelodysplastic Syndromes

**DOI:** 10.1097/HS9.0000000000000335

**Published:** 2020-01-22

**Authors:** Ángela Sánchez, Eduardo Anguita, Alberto Chaparro, Juan José Roldán-Etcheverry, Alberto López-García, Carlos Ramos-Acosta, Raluca Oancea, Diego Rodriguez-Muñoz, Susana Alemany

**Affiliations:** 1 Instituto de Investigaciones Biomédicas “Alberto Sols” Madrid, Consejo Superior de Investigaciones Científicas (CSIC-UAM) y Unidad de Biomedicina (Unidad Asociada al CSIC), Universidad de las Palmas de Gran Canaria, Spain; 2 Hematology Department, Hospital Clínico San Carlos (HCSC), Instituto de Medicina de Laboratorio (IML), Instituto de Investigación Sanitaria San Carlos (IdISSC), Department of Medicine, Universidad Complutense de Madrid (UCM). Profesor Martín Lagos s/n, Madrid, Spain; 3 Clinical Genetics Laboratory, Clinical Analysis Department, Instituto de Medicina de Laboratorio (IML), Hospital Clínico San Carlos, . Profesor Martín Lagos s/n, Madrid, Spain.

Myelodysplastic syndromes (MDS) are clonal hematopoietic stem/progenitor cell disorders associated with ineffective hematopoiesis and peripheral blood cytopenia(s). Mounting evidence suggests that immune system regulation plays an important role in MDS pathogenesis and progression.^1^ The Revised International Prognostic Scoring System (IPSS-R) divided MDS patients in four risk groups,^2^ in which, for simplicity's sake, very low, low, and intermediate risk groups can be united into one lower-risk MDS group, as higher-risk MDSs are closer to acute myeloid leukemia.

The dendritic cell (DC) system forms an essential interface in innate immunity and plays a fundamental role in sensing pathogens and activating adaptive immunity.^3,4^ Circulating DCs are divided in three major subtypes: conventional dendritic cells 1 (cDC1s), plasmacytoid dendritic cells (pDCs), and conventional dendritic cells 2 (cDC2s), each specialized in responding to particular pathogens and interacting with specific subsets of T cells (Supplementary Table 1). The internal characteristics of the generation of these three major types of DCs in MDS are largely unknown.

In this study, we aimed at gaining insight into the generation of DCs in MDS, with an emphasis on understanding the immunological abnormalities of this condition.

Dendritic cells usually constitute 0.1% to 0.5% of human peripheral blood leukocytes (PBLs).^5^ In MDS patients, Ma et al^6^ reported a decrease in two types of circulating DCs, differentiating pDCs, and conventional DCs, which were determined to be CD123^+^HLA-DR^+^CD14/CD16^dim/neg^ and CD33^+^HLA-DR^+^CD14/CD16^dim/neg^, respectively. To further explore these data, the three circulating DCs types, cDC1s, pDCs, and cDC2s were analyzed in 19 lower-risk MDS patients, and in 32 age- and sex-matched controls by flow cytometry, using a strategy in which all three DC types can be distinguished (Fig. 1A and Supplementary Methods). The lower-risk MDS patients studied are described in Supplementary Tables 2 and 3. Patients with MDS showed a significant decrease in circulating cDC1s, pDCs, and cDC2s compared to controls, with a 70% diminution in the absolute number of each of the three types of DCs (Fig. 1B). The percentages of cDC1s, pDCs, and cDC2s within the PBLs of the patients decreased with respect to the controls by a 69.3% (p = 0.0019), 58.9% (p = 0.0027) and 61.8% (p = 0.0062), respectively; despite the fact that the concentration of the PBLs was lower in MDS patients respect to the controls (Supplementary Table 3). To note, the 3 different types of circulating DCs showed similar forward scatter area (FSC-A) and side scatter area (SSC-A) values, both in controls and MDSs (data not shown). The reduction in all 3 circulating DC types in MDS patients yielded a combined concentration of 2954 ± 568 DCs/ml, which represent the 0.063 ± 0.009% of the PBLs. In the control group, the total circulating DCs represented 0.166 ± 0.017% of the PBLs with a concentration of 9718 ± 1067 DCs/ml. All these data reveal the limited number of all three types of circulating DCs in lower-risk MDS.

**Figure 1:**
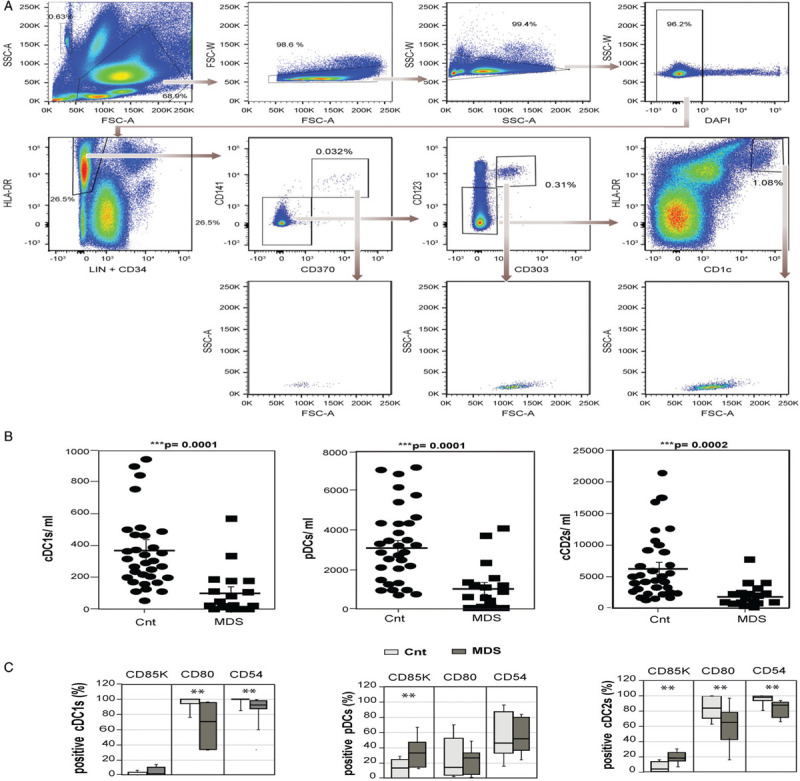
**Lower-risk MDS circulating cDC1s, pDCs, and cDC2s are reduced and abnormal**. (A) Gating strategy to identify circulating cDC1s, pDCs, and cDC2s. Single live circulating leukocytes were plotted for HLA-DR vs lineage (Lin) (CD3, CD19, CD20, and CD56) and CD34. HLA-DR^+^Lin^-^CD34^-^ cells were then gated on CD141 and CD370. Then, CD141^+^CD370^+^ cells were identified as cDC1s. CD141^-^CD370^-^ cells were further plotted according to their CD123 and CD303 expression. CD123^+^ CD303^+^ cells were identified as pDCs. CD123^-^CD303^-^ cells were subdivided based on their HLA-DR and CD1c expression, and HLA-DR^high^CD1c^+^ cells were identified as cCD2s. (B) Charts showing the amount of peripheral blood cDC1s, pDCs, and cDC2s as indicated in control (Cnt) and lower-risk myelodysplastic syndromes (MDS) (n = 32 and 19, respectively). (C) Percentage of positive cDC1s, pDCs, and cDC2s for CD85K, CD54 CD80, CD83, and CD86 markers of control (Cnt, light gray) and lower-risk MDS patients (MDS, dark gray) in peripheral blood; (n = 12 and 8, respectively). (B,C) Two-tailed Student *t* test was used for comparisons between groups. Mean ± SEM; ^∗^p < 0.05, ^∗∗^p < 0.01, ^∗∗∗^p < 0.001.

To study the status of the different circulating DCs in lower-risk MDS patients, we studied the expression of several CD markers in cells from 8 patients and 12 controls (Supplementary Table 4): CD85k (ILT-3) a tolerogenic marker, the costimulatory CD80, expressed mainly in late maturated DCs, and CD54, a marker of adhesion capacity.^7,8^ Hardly any CD85k expression could be detected in control and MDS circulating cDC1s, whereas the expression of this marker was clearly increased in MDS pDCs (10%–65%) respect to control pDCs (0%–25%). MDS cDC2s were also mainly negative for CD85k (0%–17%) and 5%–25% of MDS cDC2s were CD85K positive (Fig. 1C). These data indicate that, in lower-risk MDS, pDCs and DC2s display a tolerogenic status, which is clearly diminished in control DCs. Almost all control cDC1s (75%–100%) and 65%–100% of control cDC2s were positive for CD80, while lower-risk MDS cDC1s and cDC2s presented a decreased expression of CD80 (Fig. 1C). Besides, the CD80 mean fluorescence intensity (MFI) was higher in control CD80^+^cDC1s and CD80^+^cDC2s compared to their MDS cell counterparts (cDC1s: control 7484 ± 1054, MDS 4122 ± 811 p = 0.03; cDC2s: control 3140 ± 391, MDS cDC2s 1772 ± 637 p = 0.004). pDCs are not as good antigen-presenting cells as cDCs (Supplementary Table 1). Consistently, both control and MDS pDCs display a lower expression CD80, with low MFI values, when compared to cDCs. These data indicate that the in vivo cDCs antigen-presentation to T cells could be impaired in lower-risk MDS. Almost all control cDC1s and cDC2s were positive for CD54 and its expression decreased in a significant manner in both lower-risk MDS cDC1s and cDC2s. Besides, the MFI was again significantly higher in control cells (cDC1s: control 5707 ± 390, MDS 3774 ± 235 p = 0.001; cDC2s: control 4028 ± 3291, MDS 2649 ± 637 p = 0.043). The percentage of CD54 expression in control and lower-risk MDS tended to be similar, but the MFI was 1.8-fold higher in control pDCs (control 5018 ± 364, MDS pDCs 2835 ± 274 p = 0.002) (Fig. 1C). These data imply that another quality of DCs, their adhesion capacity, could be impaired in vivo in lower-risk MDS patients.

Both monocytes and DCs are closely related in the hematopoietic hierarchy, as they share the same lineage-restricted monocyte dendritic progenitors (MDPs). Monocytes under certain conditions are able differentiate into mono-DCs. Other groups have shown that the in vitro generation of both mono-DCs from MDS circulating monocytes and DCs from MDS bone marrow (BM) CD34^+^ cells is impaired in MDS.^9,10^ Moreover, MDS mono-DCs, and MDS DCs generated in vitro from MDS patients show a poor endocytic capacity, impaired cytokine production and a deficient induction of T-cells proliferation.^6,9,11^ These in vitro data agree with our findings, which suggest an altered acting capacity in vivo of circulating DCs in lower-risk MDS patients, besides the previously described reduction of the three circulating DCs.

To better understand the origin of this DC reduction in lower-risk MDS blood, we analyzed the DC lineage-restricted cell progenitors encompassed within the BM CD34^+^CD38^+^ cell compartment by flow cytometry (10 patients and 10 controls, Supplementary Fig. 1, Supplementary Table 5). First, we observed that within the total BM CD34^+^CD38^+^ cell population, the percentage of CD10^high^CD34^+^CD38^+^ cells, that includes different lymphoid-restricted progenitors,^4,5,12^ was reduced by 80% in the MDS group (Fig. 2A). CD10^−^CD34^+^CD38^+^ progenitor cells were gated according to their CD45RA to distinguish the CD45RA^−^CD10^−^CD34^+^CD38^+^ cell population, which includes the megakaryocyte erythroid cell progenitors (MEPs) and the common myeloid cell progenitors (CMPs), from which all myeloid cells derive; and the CD45RA^+^CD10^−^CD34^+^CD38^+^ cells (Supplementary Methods, Supplementary Fig. 1A), which include 3 different myeloid progenitors: granulocyte monocyte dendritic cell progenitors (GMDPs), MDPs, and common dendritic cell progenitors (CDPs).^5,12^ The GMDPs, which originate directly from CMPs, differentiate into the granulocytic lineage and also progress to MDPs. MDPs differentiate into the monocytic cell lineage and also give rise to CDPs directly. CDPs are the most differentiated cell type that only generates the 3 circulating dendritic cells, but no other cell types.^3,5,12^ Lower-risk MDS patients showed an increased ratio of CD45RA^−^CD10^−^CD34^+^CD38^+^ with respect to CD45RA^+^CD10^−^CD34^+^CD38^+^ cells compared to controls (Fig. 2B,C). Analysis of the percentage of GMDPs, MDPs, and CDPs within the CD45RA^+^CD10^−^CD34^+^CD38^+^ cell compartment revealed a similar percentage of GMDPs and MDPs in both patients and controls (Fig. 2D), which yielded a decrease of these 2 cell populations with respect to the total number of CD34^+^CD38^+^ cells in the BM of lower-risk MDS patients (Fig. 2E). However, the percentage of CDPs increased 1.7 fold within the CD45RA^+^CD10^−^CD34^+^CD38^+^ cell compartment in the MDS patients with respect to the controls (Fig. 2D), displaying a similar percentage of CDPs within the CD34^+^CD38^+^ cells in both the MDS and the control groups (Fig. 2E). Similar data were obtained when CDPs were identified by a different gating strategy: CD34^+^CD38^+^→CD123^high^CD34^+^CD38^+^→CD10^−^CD45RA^+^CD123^high^CD34^+^CD38^+^ cells (data not shown). CDPs are the major cell population expressing high levels of CD123 within the CD34^+^CD38^+^ cell compartment,^5^ and, in agreement with the similar percentage of CDPs in this cell population, CD123 was similarly expressed in CD34^+^CD38^+^ cells from both the MDS and the control groups, with values of 5.9% ± 0.67 and 5.07% ± 0.65, respectively (p = 0.35) (Supplementary Fig. 1B).

**Figure 2:**
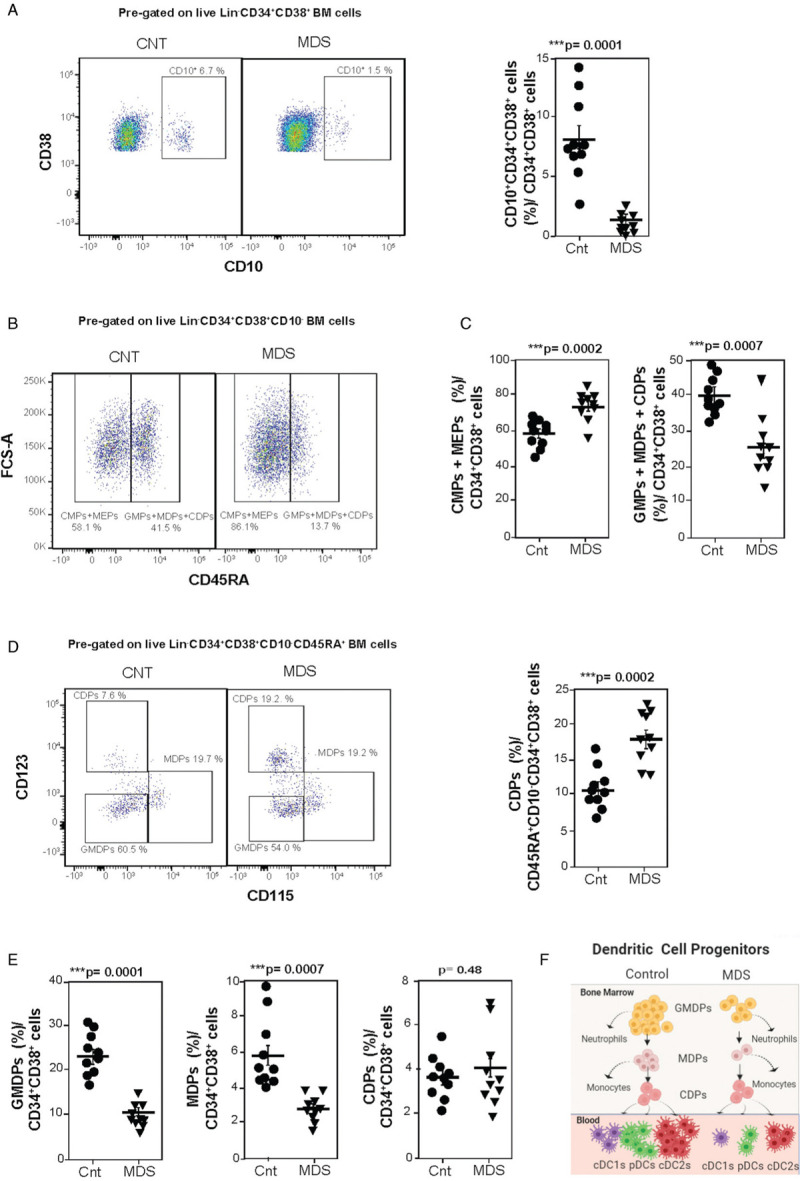
**CDPs increase with respect to their precursors, GMDPs and MDPs, in bone marrow of lower-risk MDS patients**. (A) Representative flow cytometry plots (left) and dots chart (right) displaying the percentage of CD10+ cells within CD34^+^CD38^+^ cell population in BM of control (Cnt) and lower-risk MDS patients (MDS). (B) Representative plots showing the percentage of CD45RA^-^ (CMPs and MEPs) and CD45RA^+^ (GMDPs, MDPs, and CDPs) cell populations within CD10^-^CD34^+^CD38^+^ cells. (C) Percentage of CMPs plus MEPs (left hand panel) and GMDPs together with MDPs and CDPs (right), within CD34^+^CD38^+^ cells, from samples described in A. (D) Left, representative graphs showing the percentage of GMDPs, MDPs, and CDPs within Lin^-^CD45RA^+^CD10^-^CD34^+^CD38^+^ cells in BM of control and lower-risk MDS patients. Right, percentage of CDPs within CD45RA^+^CD10^-^CD34^+^CD38^+^ cells in BM from samples described in A. (E) Percentage of GMDPs, MDPs, and CDPs within CD34^+^CD38^+^ cells from samples described in A. (F) Model of DC-poiesis in control and lower-risk MDS patients, the number of cells depicted symbolize the relative proportion of the indicated cell populations in human lower-risk MDS and control bone marrow and peripheral blood. CDPs is the only cell population that is not reduced comparing lower-risk MDS and controls. DC, dendritic cell; GMDPs, granulocyte monocyte dendritic cell progenitors; MDPs, monocyte dendritic progenitors; CDPs, common dendritic cell progenitors. (A-E) Mean ± SEM; n = 10 in both groups. Two-tailed Student *t* tests were used for comparisons between 2 groups, ^∗^p < 0.05, ^∗∗^p < 0.01, ^∗∗∗^p < 0.001.

In lower-risk MDS, previous studies, focused on the hematopoiesis of other myeloid cells such as neutrophils and monocytes, have shown an increased percentage of CMPs,^13^ and a decrease of GMPs within the BM CD34^+^CD38^+^ cell compartment of these patients.^14^ Our data, focused on the progenitors of circulating DCs, indicate a relative accumulation of CDPs with respect to their direct precursors, GMDPs and MDPs, within BM CD34^+^CD38^+^ cells of lower-risk MDS patients. This, together with the severe reduction of circulating cDC1s, pDCs, and cDC2s, suggests an inefficient progression of CDPs towards these three DC types in lower-risk MDS (Fig. 2F). A recent study indicated that BM conventional DCs show immature transcriptional and functional characteristics compared to their circulating counterparts.^15^ The analysis of this particular cell population in MDS may be assessed in future studies.

Lower-risk MDS patients peripheral blood DCs are the circulating myeloid cells that show a greater reduction (Supplementary Table 2). Each dendritic cell type analyzed here plays a specialized immune function (Supplementary Table 1) and the severe deficiency of the three types of circulating DCs in the lower-risk MDS patients, as well as their impaired status, might contribute, together with the deficiency in other myeloid cells, to the recurrent infections that affected the MDS patients studied here (Supplementary Results and Supplementary Table 2), which indeed is a characteristic of this disease.^16^

In conclusion, our data show lower-risk MDS patients display an increased susceptibility to infections, an inadequate DC-poiesis, and a deficient number of abnormal circulating DCs.

## Supplementary Material

Supplemental Digital Content
