# Supplementary material for: Abnormal Dendritic Cell-poiesis in Patients With Lower-risk Myelodysplastic Syndromes
Source: Hemasphere. 2020 Jan 22;4(1):e335. doi: 10.1097/HS9.0000000000000335 (PMC7000478; doi:10.1097/HS9.0000000000000335)
Supplement: Supplemental Digital Content [file hs9-4-e335-s001.docx]

**Supplementary Methods**

**Patient samples**

Peripheral blood (PB) and bone marrow (BM) samples were collected in EDTA containing tubes; they were taken from unselected patients, scheduled at Hospital Clínico San Carlos for cytology and cytogenetic studies and diagnosed with lower-risk MDS -see Supplementary Tables. Controls consisted of PB from 22 healthy individuals together with 10 PB and BM samples from patients with no myelopoietic disorders (bone marrow extraction from healthy individuals was not allowed for ethical reasons): 1 iron deficiency anemia, 1 hypersplenism, 1 *Helicobacter pylori*-associated thrombocytopenic purpura, 4 with non-Hodgkin lymphoma without BM infiltration -one thyroid marginal zone lymphoma, one primary bone tibia diffuse large B-cell lymphoma, one nodal diffuse large B-cell lymphoma at diagnosis and one nodal diffuse large B-cell lymphoma in complete remission-, 3 subjects diagnosed with immune thrombocytopenic purpura -one of them with anemia of chronic disease and another one with iron deficiency anemia- (Supplementary Tables). All samples have been collected according to institutional ethic procedures; appropriate patient consent and information have been performed according to the procedures stipulated by the Ethic Committee of Hospital Clínico San Carlos. Patients were classified using standard methods into the Revised International Prognostic Scoring System (IPSS-R) according to their blood counts, bone marrow morphology and cytogenetic results including karyotype, FISH and/or Multiplex Ligation-dependent Probe Amplification (MLPA) (SALSA MLPA P414 MDS probemix, MRC, Holland).

**Flow cytometry**

Blood samples (300 μl) were treated for 20 min with *Versalyse* lysing solution (A0977, Beckman Coulter) at room temperature and washed twice with 2% BSA in PBS. Cells were stained 20 minutes at room temperature with anti-human antigen presenting cell cocktail APC (containing antibodies against CD3, CD19, CD20, and CD56; 363601, Biolegend), HLA-DR APC-Cy7 (L243, Biolegend), CD141 (BDCA-3) Viobright or APC-Vio770 (REA674, Miltenyi Biotec), CD370 (CLEC9A/DNAGR1) PE (8F9, Biolegend), CD123 (IL-3Rα) brilliant violet 510 (6H6, Biolegend), CD303 (BDCA-2) PerCP-Cy5 (201A, Biolegend,) and CD1c (BDCA-1) PE-Cy7 or Pacific Blue (L161, Biolegend); and when indicated CD85K PE-Cy7 (ZM4.1, Biolegend), CD54 (ICAM-1) FITC (HCD54, Biolegend), or CD80 PE-Cy7 ([16-10A1](https://www.biolegend.com/en-us/search-results?Clone=16-10A1), Biolegend). Subsequently, cells were washed once with 2 % BSA in PBS. Live and dead cells were separated by adding DAPI ([D9542](http://www.sigmaaldrich.com/catalog/product/sigma/d9542?lang=es&region=ES), Sigma-Aldrich) or SYTOX Green (S7020, Life Technologies) 5 min before FACS analysis. Fresh human BM obtained by centrifugation at 1000 G on ficoll gradient (Lymphoflot; 824012, BIO-RAD) was treated for 10 min with *Versalyse* lysing solution at room temperature and washed with 2 % BSA in PBS. Cells were then stained with the anti-human antigen presenting cell cocktail APC (CD3, CD19, CD20 and CD56; 363601, Biolegend), CD10 (CALLA) PE-Cy5 (A07761 Beckman Coulter), CD11b APC (M1/70, Biolegend), CD34 PE-Cy7 (4H11, eBioscience), CD38 eFluor 450 (HB7, eBioscience), CD115 PE (9-4D2-1E4, Biolegend), CD123 brilliant violet 510 (6H6, Biolegend) and CD45RA APC eFluor 781 (HI100, eBioscience) for 20 min at room temperature and subsequently washed once with 2 % BSA in PBS. Live and dead cells were distinguished by adding SYTOX Green before FACS analysis. Unstained cells were used as a negative control to establish the flow cytometer voltage settings, and single-colour positive controls were used to adjust compensation. The absolute number of cells was calculated by adding **Perfect-Count Microspheres** (CYT-PCM-100, Cytognos) to the flow cytometry samples. The flow cytometry data were acquired using a FACSCanto II and analysed with FACSDiva (Becton and Dickinson) or FlowJo software.

**Statistical analysis**

The results are expressed as means ± SEM. Two-tailed Student's *t*-tests were used for comparisons between groups using the GraphPad software. Differences between the groups were considered significant if: **p*<0.05, ***p*<0.01, or ****p*<0.001.

**Supplementary Results**

Medically attended infections attributed to bacteria were reported in eleven of the 21 patients from this study (52.38%). They suffered a total of 20 episodes in a 20-month follow-up and five of them (23.8%) required hospital admission, with a total of 11 hospitalizations during this period. On the other hand, only seven infections were reported in the 31 controls (22.58%), 9 episodes, and two of them needed to stay in the hospital (6.45%). Infection in lower-risk-MDS were: 5 bronchitis, 5 pneumonias (one produced the death of a patient), 5 urinary tract infections, 1 bacteremia, 1 cholangitis, 1 cellulitis and 2 unspecified infections. Also, one patient suffered a progressive multifocal leukoencephalopathy (PML) that needed hospitalization. Identified bacteria in lower-risk MDS were *Proteus mirabilis* (tree cases), *Escherichia coli* (twice), *Staphylococcus epidermidis* (twice), *Pseudomonas aeruginosa* (twice), *Corynebacterium amycolatum* and methicillin-resistant *Staphylococcus aureus*. Infections in control group were 2 bronchitis, 1 urinary tract infection, 1 cellulitis and 5 unspecified infections. The identified bacteria in the controls were: 1 Pseudomonas aeruginosa, 1 Citrobacter koseri and 1 Staphylococcus epidermidis.


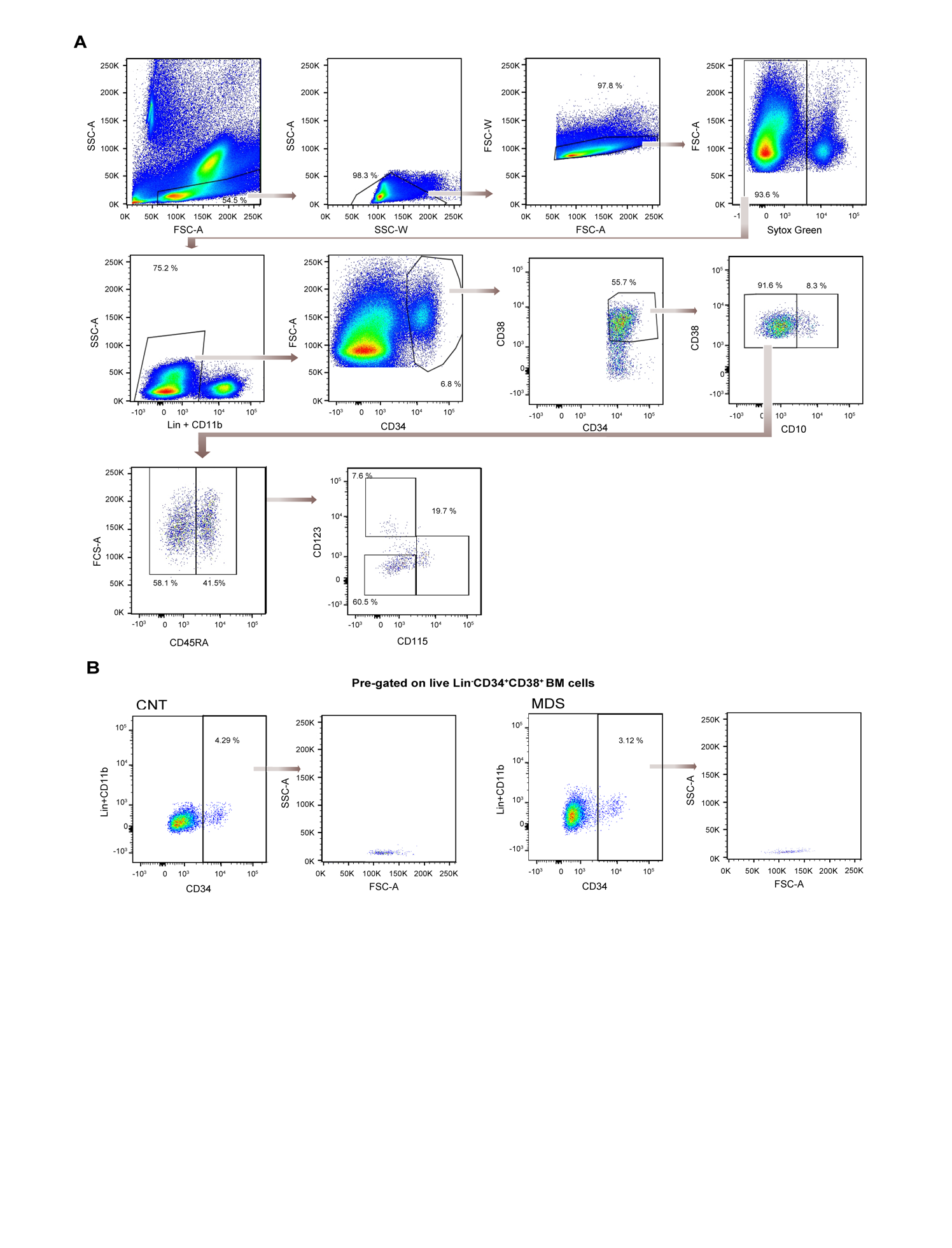


**Supplementary Figure 1.** **Gating strategy for identification of dendritic cell progenitors.** **(A)** Lineage (Lin)-negative (CD2, CD3, CD11b, CD14, CD15, CD16, CD19, CD56, CD61 and CD235a)^-^ magnetic-activated sorted single live bone marrow cells were plotted for forward scatter area (FSC-A) versus Lin (CD3, CD19, CD20 and CD56) plus CD11b. Subsequently, Lin^-^CD11b^-^ cells were further gated on FSC-A and CD34. CD34^+^ cells were further plotted for CD34 and CD38. CD34^+^CD38^+^ cells were divided on basis of their CD10 expression. CD10^-^CD34^+^CD38^+^ cells were further plotted for FCS-A and CD45RA. CD45RA^+^CD10^-^CD34^+^CD38^+^ cells were then divided in basis of their CD123 and CD115 expression. CD123^high^CD115^-^ cells were identified as common dendritic cell progenitors (CDPs), CD115^-^CD45RA^-^ cells were identified as granulocyte monocyte dendritic cell progenitors (GMDPs) and CD123^med/low/neg^CD115^+^ cells were identified as monocyte dendritic progenitors (MDPs). **(B)** Representative plots showing the percentage of CD123^+^CD34^+^CD38^+^ cells within BM CD34^+^CD38^+^ cells from control and lower-risk MDS patients, and their side scatter area (SSC-A) versus FCS-A size.

**Supplementary Table 1**

| **DC subset** | **Phenotype** | **Function** | **Ref.** |
| --- | --- | --- | --- |
| cDC1 | HLADR^+^Lin^-^CD141(BDCA3/)^+^  CD370(CLEC9A/DNGR1) ^+^ | Specialize in cross-presentation and CD8^+^ T cell priming for tumors and pathogens that do not directly infect DCs. | *(1-6)* |
| pDC | HLA-DR^+^Lin^-^CD123(IL3-R)^+^CD303(BDCA2)^+^ | Recognition of viruses, INF-α,−β production; activation of NK cells, IL-12 and IL-18; production of other cytokines | *(1, 2, 7-9)* |
| cDC2 | HLA-DR^high^Lin^-^CD1c(BDCA1)^+^ | Expert in CD4^+^ T cell priming and promoting Th17-biased; high production of interferon-γ, IL-17A, IL-17F, IL-21, and IL-22 | *(1, 2, 7, 10, 11)* |

**Human circulating dendritic cell subsets.** Lin, Lineage= CD3, CD19, CD20, and CD56

**References**

1. Villani AC, Satija R, Reynolds G, Sarkizova S, Shekhar K, Fletcher J, et al. Single-cell RNA-seq reveals new types of human blood dendritic cells, monocytes, and progenitors. Science. 2017 Apr 21;356(6335).

2. Merad M, Sathe P, Helft J, Miller J, Mortha A. The dendritic cell lineage: ontogeny and function of dendritic cells and their subsets in the steady state and the inflamed setting. Annu Rev Immunol. 2013;31:563-604.

3. Cohn L, Chatterjee B, Esselborn F, Smed-Sorensen A, Nakamura N, Chalouni C, et al. Antigen delivery to early endosomes eliminates the superiority of human blood BDCA3+ dendritic cells at cross presentation. J Exp Med. 2013 May 6;210(5):1049-63.

4. Audiger C, Rahman MJ, Yun TJ, Tarbell KV, Lesage S. The Importance of Dendritic Cells in Maintaining Immune Tolerance. J Immunol. 2017 Mar 15;198(6):2223-31.

5. Persson EK, Uronen-Hansson H, Semmrich M, Rivollier A, Hagerbrand K, Marsal J, et al. IRF4 transcription-factor-dependent CD103(+)CD11b(+) dendritic cells drive mucosal T helper 17 cell differentiation. Immunity. 2013 May 23;38(5):958-69.

6. Noubade R, Majri-Morrison S, Tarbell KV. Beyond cDC1: Emerging Roles of DC Crosstalk in Cancer Immunity. Front Immunol. 2019;10:1014.

7. Collin M, Bigley V. Human dendritic cell subsets: an update. Immunology. 2018 May;154(1):3-20.

8. Swiecki M, Colonna M. The multifaceted biology of plasmacytoid dendritic cells. Nat Rev Immunol. 2015 Aug;15(8):471-85.

9. Zhang H, Gregorio JD, Iwahori T, Zhang X, Choi O, Tolentino LL, et al. A distinct subset of plasmacytoid dendritic cells induces activation and differentiation of B and T lymphocytes. Proc Natl Acad Sci U S A. 2017 Feb 21;114(8):1988-93.

10. Leal Rojas IM, Mok WH, Pearson FE, Minoda Y, Kenna TJ, Barnard RT, et al. Human Blood CD1c(+) Dendritic Cells Promote Th1 and Th17 Effector Function in Memory CD4(+) T Cells. Front Immunol. 2017;8:971.

11. Segura E, Villadangos JA. Antigen presentation by dendritic cells in vivo. Curr Opin Immunol. 2009 Feb;21(1):105-1

**Supplementary Table 2**

| ID | Age (Y) | Gen (M/F) | WHO type | % Blast | Hb (g/dL) | Neutro (10^9^/L) | Plt (10^9^/L) | Karyotype | MLPA | IPSS-R  (score) | Infections  (20 months) | Hospitalization | Pathogen |
| --- | --- | --- | --- | --- | --- | --- | --- | --- | --- | --- | --- | --- | --- |
| 1^$^ | 62 | F | del(5q) | <2% | 13.1 | 1.1 | 97 | 46,XX,del(5q31) | del(5q) | VL (1.5) | NO | None | None |
| 2^$^ | 79 | F | del(5q) | <2% | 12.6 | 3.6 | 99 | 46,XX,del(5q31) | del(5q) | VL (1.5) | Yes, U | None | UD |
| 3^$^ | 83 | F | del(5q) | 3-5% | 10.3 | 0.4 | 34 | 46,XX,del(5q31) | del(5q) | Int (3.5) | NO | None | None |
| 4^$^ | 64 | M | MLD | <2% | 11.1 | 1.8 | 208 | 46,XY | Normal | VL (1) | NO | None | None |
| 5^$^ | 77 | M | RS-MLD | <2% | 9.1 | 6.4 | 230 | No metaphases | +8 | L (3) | NO | None | None |
| 6^$^ | 85 | M | RS-MLD | <5% | 8.7 | 6.7 | 202 | No metaphases | +8 | L (3) | NO | None | None |
| 7^$^ | 72 | M | EB-2 | 10-15% | 13 | 2.1 | 80 | 45,X,-Y | del(Y) | Int (3.5) | Cellulitis & UTI | None | 1. UD  2. *P. mirabilis* |
| 8^$$^ | 76 | M | MLD | <2% | 10.9 | 1 | 85 | No metaphases | Normal | VL (1.5) | NO | None | None |
| 9^$$^ | 67 | M | RS-MLD | <2% | 12 | 2.5 | 329 | 46,XY | Normal | VL (1) | NO | None | None |
| 10^$$^ | 72 | F | RS-MLD | <2% | 11.2 | 3.7 | 321 | 46,XX | Normal | VL (1) | UTI | None | 1. *E. coli* |
| 11^$$^ | 57 | F | MLD | 3-5% | 9.6 | 0.3 | 287 | 47,XX,+8 | +8 | Int (4.5) | Bronchitis | None | UD |
| 12^$$^ | 77 | F | RS-MLD | <2% | 11.5 | 2.4 | 176 | No metaphases | Normal | VL (1) | NO | None | None |
| 13^#^ | 85 | M | del(5q) | <2% | 10.7 | 1.3 | 79 | 46,XY,del(5)(q31) | del(5q) | VL (1.5) | Bronchitis | None | UD |
| 14^#^ | 79 | F | MLD | <2% | 8.3 | 2 | 63 | No metaphases | Normal | L (2.5) | Cholangitis | 1 | UD |
| 15^#^ | 68 | F | EB-1 | 5-10% | 13.7 | 3.2 | 155 | 46,XX | Normal | L (3) | Pneumonia | 1 | *C. amycolatum* |
| 16^#^ | 74 | M | MLD | <2% | 12.1 | 4.4 | 101 | 46,XY | +3q; del(5q)*; del(7q)* | L (3) | NO | None | None |
| 17^#^ | 79 | M | MLD | <2% | 6.7 | 1.4 | 138 | 46,XY | Normal | L (2.5) | Bronchitis, Pneumonia (x4) & PML | 6** | 1. *P. aeruginosa*  2. UD  3. MRSA  4. P*. aeruginosa*  5. *S. epidermidis* |
| 18^#^ | 87 | M | MLD | <2% | 11.7 | 2.7 | 72 | No metaphases | Normal | VL (1.5) | NO | None | None |
| 19^#^ | 86 | M | MLD | <2% | 8.8 | 1.1 | 310 | 47,XY,+8 | +8 | L (3) | Bronchitis & UTI | 2 | UD |
| 20^+^ | 85 | M | MLD | <2% | 12.3 | 1.4 | 43 | 46,XY | Normal | L (2) | Bronchitis, Bacteremia & UTI | 2 | 1. UD  2. *S. epidermidis*  3. *E. coli* |
| 21^++^ | 76 | F | RS-MLD | 3-5% | 9.2 | 2.4 | 160 | 46,XY | Normal | L (2) | UTI | None | *P. mirabilis* |

**Characteristics of the lower-risk MDS patients.** ID, identification number; Y, years; Gen, gender; M, male; F, female; WHO, World Health Organization; Hb, hemoglobin; Neutro, neutrophils; ptl, platelets; MLPA, multiplex ligation-dependent probe amplification; FISH, fluorescence in situ hybridization; IPSS-R, revised international prognostic scoring system; MLD, myelodysplastic syndrome (MDS) with multilineage dysplasia; RS-MLD, MDS with ring sideroblasts and multilineage dysplasia; EB, MDS with excess blasts; VL, very low; Int, intermediate; L, low; U, unspecified; UTI, urinary tract infection; PML, progressive multifocal leukoencephalopathy; UD, undetermined; MRSA, methicillin-resistant *Staphylococcus aureus*. ^$^Patients analyzed for DCs quantification in peripheral blood, circulating DCs surface markers and bone marrow DCs-poiesis; ^$$^Patients analyzed for DCs quantification in peripheral blood and bone marrow DCs-poiesis; ^#^Patients analyzed for DCs quantification in peripheral blood only; ^+^Patients analyzed in bone marrow only; ^++^Patients analyzed for circulating DCs surface markers only. *Interstitial (abnormalities in this patient in 15-20% of the sample). **Including a hospitalization with PML.

**References**

1 Greenberg PL, Tuechler H, Schanz J, Sanz G, Garcia-Manero G, Sole F, Bennett JM, Bowen D, Fenaux P, Dreyfus F, Kantarjian H, Kuendgen A, Levis A, Malcovati L, Cazzola M, Cermak J, Fonatsch C, Le Beau MM, Slovak ML, Krieger O, Luebbert M, Maciejewski J, Magalhaes SM, Miyazaki Y, Pfeilstocker M, Sekeres M, Sperr WR, Stauder R, Tauro S, Valent P, Vallespi T, van de Loosdrecht AA, Germing U, Haase D. Revised international prognostic scoring system for myelodysplastic syndromes. Blood. 2012;120:2454-2465

**Supplementary Table 3**

|  | **CONTROLS** | **LOWER-RISK MDS** | **p-value** |
| --- | --- | --- | --- |
| **Age** | 77.4 ± 2.53 | 75.2 ± 1.97 | ns |
| **Gender M(%)/F** | 56.3 | 57.9 |  |
| **PBLs (x10^9^)/L** | 6.2 ± 0.31 | 4.7 ± 0.57 | * |
| **Neutrophils (x10^9^)/L** | 3.8 ± 0.27 | 2.5 ± 0.41 | ** |
| **Lymphocytes (x10^9^)/L** | 1.6 ± 0.15 | 1.5 ± 0.19 | ns |
| **Monocytes (x10^9^)/L** | 0.5 ± 0.03 | 0.4 ± 0.07 | ns |
| **Hemoglobin g/dL** | 13.7 ± 0.40 | 10.8 ± 0.43 | *** |
| **Platelets (x10^9^)/L** | 190.0 ± 14.23 | 161.4 ± 22.00 | ns |

**Demographic characteristics, blood counts and infections of control and lower-risk MDS groups studied for DCs quantification in peripheral blood.** M, male; F, female; PBLs, peripheral blood leukocytes; ns, not significant. Data show the mean ± SEM; controls n= 32; lower-risk MDS n= 19. Two-tailed Student's *t*-tests were used for comparisons between two groups, **p*<0.05, ****p*<0.001.

**Supplementary Table 4**

|  | **CONTROLS** | **LOWER-RISK MDS** |  |
| --- | --- | --- | --- |
| **Age** | 70.3 ± 4.17 | 76.6 ± 2.73 | ns |
| **Gender M(%)/F** | 50.0 | 50.0 |  |
| **PBLs (x10^9^)/L** | 5.7 ± 0.37 | 5.7 ± 0.96 | ns |
| **Neutrophils (x10^9^)/L** | 3.4 ± 0.29 | 3.0 ± 0.80 | ns |
| **Lymphocytes (x10^9^)/L** | 1.6 ± 0.19 | 1.9 ± 0.19 | ns |
| **Monocytes (x10^9^)/L** | 0.5 ± 0.05 | 0.6 ± 0.16 | ns |
| **Hemoglobin g/dL** | 14.6 ± 0.52 | 10.4 ± 0.76 | *** |
| **Platelets (x10^9^)/L** | 183.6 ± 16.00 | 151.9 ± 25.92 | ns |

**Demographic characteristics, blood counts and infections of control and lower-risk MDS groups** **for the analysis of circulating DCs surface markers.** M, male; F, female; PBLs, peripheral blood leukocytes; ns, not significant. Data show the mean ± SEM; controls n= 12; lower-risk MDS n= 8. Two-tailed Student's *t*-tests were used for comparisons between two groups, **p*<0.05, ****p*<0.001.

**Supplementary Table 5**

|  | **CONTROLS** | **LOWER-RISK MDS** | **p-value** |
| --- | --- | --- | --- |
| **Age** | 72.7 ± 2.88 | 73.9 ± 2.85 | ns |
| **Gender M(%)/F** | 70.0 | 60.0 |  |
| **PBLs (x10^9^)/L** | 5.2 ± 0.57 | 5.6 ± 0.91 | ns |
| **Neutrophils (x10^9^)/L** | 2.9 ± 0.43 | 3.0 ± 0.68 | ns |
| **Lymphocytes (x10^9^)/L** | 1.5 ± 0.34 | 1.9 ± 0.23 | ns |
| **Monocytes (x10^9^)/L** | 0.5 ± 0.05 | 0.5 ± 0.11 | ns |
| **Hemoglobin g/dL** | 12.5 ± 0.78 | 10.9 ± 0.43 | ns |
| **Platelets (x10^9^)/L** | 147.0 ± 27.21 | 198.0 ± 31.35 | ns |

**Demographic characteristics, blood counts and infections of control and lower-risk MDS groups** **for bone marrow analysis.** M, male; F, female; PBLs, peripheral blood leukocytes; ns, not significant. Data show the mean ± SEM; controls n=10; lower-risk MDS n=10. Two-tailed Student's *t*-tests were used for comparisons between two groups, **p*<0.05, ****p*<0.001.
